# Local non-equilibrium thermodynamics

**DOI:** 10.1038/srep07832

**Published:** 2015-01-16

**Authors:** Lee Jinwoo, Hajime Tanaka

**Affiliations:** 1 Department of Mathematics, Kwangwoon University, 20 Kwangwoon-ro, Nowon-gu, Seoul 139-701, Korea; 2 Institute of Industrial Science, University of Tokyo, 4-6-1 Komaba, Meguro-ku, Tokyo 153-8505, Japan

## Abstract

Local Shannon entropy lies at the heart of modern thermodynamics, with much discussion of trajectory-dependent entropy production. When taken at both boundaries of a process in phase space, it reproduces the second law of thermodynamics over a finite time interval for small scale systems. However, given that entropy is an ensemble property, it has never been clear how one can assign such a quantity *locally*. Given such a fundamental omission in our knowledge, we construct a new ensemble composed of trajectories reaching an individual microstate, and show that locally defined entropy, information, and free energy are properties of the ensemble, or trajectory-independent true thermodynamic potentials. We find that the Boltzmann-Gibbs distribution and Landauer's principle can be generalized naturally as properties of the ensemble, and that trajectory-free state functions of the ensemble govern the exact mechanism of non-equilibrium relaxation.

Statistical mechanics provides physical interpretations of entropy and free energy that are macro-state functions (*i.e.*, functions defined on a domain of the phase-space points of a system, and thus inevitably non-local in character), and sets bounds on permissible processes expressed as path functions like heat and work, where a path is defined as a trajectory of macrostates of a system^1^. As a system gets smaller, the effect of fluctuations becomes significant, yet modern theory^2^,^3^,^4^ provides permissible distributions of fluctuating path functions in the form of beautiful equalities^5^,^6^,^7^,^8^,^9^,^10^. Modern theory has identified energetics^11^ and entropy production on the level of individual trajectories^12^, and has linked path functions to properties of macrostates^13^, as verified experimentally^14^,^15^,^16^,^17^,^18^,^19^. The relationship between classical and modern approaches is schematically drawn in Fig. 1.

Despite these successes in non-equilibrium theories, however, still lacking is a unified framework analogous to classical thermodynamics^20^. It is important to note that contrary to the classical cases, a path in the modern theory is defined as a trajectory of phase-space points of a system. In general, the modern theory has been built effectively upon quantities defined locally (*i.e.*, functions defined at each phase-space point). For example, a local form of information theoretic Shannon entropy, so called stochastic entropy, is defined as − ln *p*(*x*, *t*), where *p*(*x*, *t*) is the phase-space density of a microstate *x* of a system. Such a local form is necessary for boundary conditions of a process (a path) to recover the second law of thermodynamics over a finite time interval for small scale systems^8^,^12^. Then we immediately encounter a serious conceptual difficulty. As a component of the second law, the entropy should be a property of an ensemble. However, it has not been clear at all how such an ensemble property as entropy could be assigned *locally*, or independently of a trajectory. This difficulty in the notion of locality is also closely associated with a classical view of difficulties of non-equilibrium thermodynamics; the ill-defined notion of a macrostate during dynamic evolution of a system. It is thus difficult to apply the language of equilibrium thermodynamics, most importantly the argument of counting, to non-equilibrium problems.

Without counting, for both equilibrium and non-equilibrium situations, we have no way to even imagine what entropy is. We remove this fundamental difficulty by constructing a new ensemble that is *local* in space and time, and is well-defined even in dynamic evolution of a system. Specifically, we count the accessible number of paths to a microstate and consider all trajectories to a microstate in non-equilibrium as an ensemble of the state. We, then, show that local entropy, similarly defined local information and local free energy are properties of the ensemble, and relate functions within the ensemble to state functions of the ensemble which have been concealed behind the integrated-out forms of previous theories.

## Theoretical Framework

**Figure 1:**
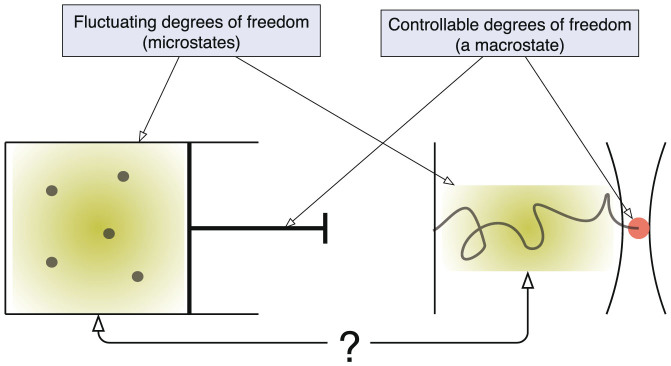
A universal frame of thermodynamics and a fundamental question. Thermodynamics is a theory for ensembles that are composed of a large number of fluctuating degrees of freedom. An ensemble is constrained by a macrostate *λ*. In equilibrium thermodynamics, *λ* varies in a quasi-static manner. In the modern context, the theory considers arbitrary time-varying processes. Two typical systems in both approaches are shown schematically. On the left, gas particles are fluctuating in a cylinder. The macrostate may be specified by the temperature, the volume of the cylinder, and the number of particles. On the right, a polymer chain is fluctuating under the influence of optically controlled bead connected to the end of the chain. The macrostate may be specified by the temperature, and the location of the bead applying force to the molecule. A fundamental question that we investigate is how we can justify thermodynamic descriptions of fluctuating microstates.

In this article, we consider a classical stochastic system in contact with a heat bath of temperature *T*. To provide some intuitive grasp, we take as a simple example a polymer chain whose one end is fixed and the other end under an external control *λ_τ_* that may vary over time 0 ≤ *τ* ≤ *t* (see the right panel of Fig. 1). We will refer to *λ_τ_* as a macrostate (the state of a polymer chain) at time *τ*, and to the phase-space points of the polymer chain (excluding momentum variables for simplicity) as microstates. Note that, in this experiment, *λ_τ_* is the only parameter controlling the macrostate of a polymer chain.

We define a forward process as one where an external control *λ_τ_* is varied from *λ*_0_ to *λ_t_* during 0 ≤ *τ* ≤ *t* in a well-defined manner, and a corresponding reverse process 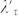 as 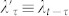. Let *l* be a space-time trajectory of the system's microstates (*i.e.*, evolving polymer configurations) during a forward process. Since *l* fluctuates, we repeat the process *λ_τ_* with appropriate initial probability density *p*(*x*, 0) over all microstates *x*. When we consider a microstate along a path *l* at time *τ*, we will denote it as *l_τ_*. We define path-dependent work done on the system by the external control during a forward process as follows: 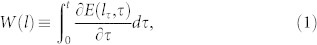
where *l* is a trajectory, *E*(*l_τ_*, *τ*) is the energy of a microstate *l_τ_* at time *τ*. Path-dependent heat transferred to the system during the same process is defined as follows: 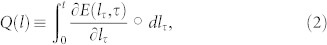
where ○ *dl_τ_* means either 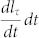 for deterministic frameworks, or a multiplication in the Stratonovich sense for stochastic frameworks. Then, for each *l*, by adding the heat and work during the forward conversion we have the first law of thermodynamics in the following form: 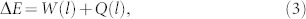
where Δ*E* = *E*(*l_t_*, *t*) − *E*(*l*_0_, 0).

**Figure 2:**
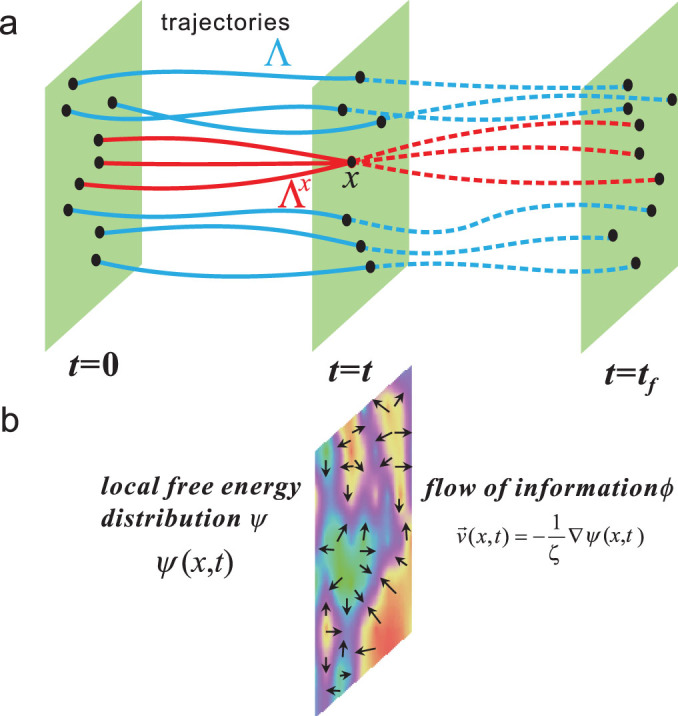
An evolution of a system's microstates. (a), A microstate as an ensemble of trajectories to a phase-space point. Each space in green represents the phase space of a system (excluding momentum variables for simplicity) at time *t*. Each trajectory shown schematically represents a stochastic evolution of the phase-space points. Among all possible trajectories Λ*^x^*, we focus on those paths (Λ*^x^*: thick red lines) that reach a specific point *x* in the phase space at time *t* = *t*. (b), Schematic figure of the distribution of *ψ*(*x*, *t*) at time *t* = *t* in the phase space, where the information flow is to be induced in the directions indicated by black arrows. The information flow not only causes the convective transport of the information *φ*(*x*, *t*), but also its divergence provides the net change of *φ*(*x*, *t*) (see Eq. (16)).

Let Λ be the set of all space-time trajectories of a system connecting times 0 and *t*, and Λ*^x^* be those paths that reach a phase space point *x* at time *t* from the past (see Fig. 2). There is a natural projection *π* from Λ*^x^* onto *x* such that *π*(Λ*^x^*) = *x*, and then each microstate *x* can be regarded as an ensemble with conceptual rigour. This is because *π*^−1^(*x*) includes an *infinite* number of stochastic trajectories reaching *x*. This indicates that *thermodynamic* quantities can be defined for *x*.

Our results will be built upon two assumptions. Firstly, we assume that the probability of path *l* ∈ Λ is represented as 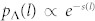 for some function *s*. The explicit form of *s* depends on the type of model. For example, it would be the stochastic action with an initial condition derived by Onsager for stochastic models^21^, the Hamiltonian of a system^20^, or a bath at time 0 for deterministic frameworks^22^. Based on this, we develop the following idea: Since each path may not be equally probable, we assign each path a weight of the form *g*(*l*) ≡ *e*^−*s*(*l*)^ so that less probable paths would be less counted. Let Ω(*X*) be the *accessible number* (or the total weight) of paths in a sample space *X*, then, we have 
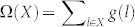
. In this framework, every *unit* path, a collection of paths of which total weight is 1, is equally probable, and the probability of a microstate (*e.g.*, a chain configuration) *x* at time *t* would be simply the ratio of the accessible number (or the total weight) of trajectories that pass *x* at time *t* to that of all possible trajectories, *i.e.*, Ω(Λ*^x^*)/Ω(Λ). This new framework provides us with a basis for “counting” analogous to classical entropy.

Secondly, we assume microscopic reversibility^8^,^22^, proven by Jarzynski in the Hamiltonian framework^22^, and valid in various stochastic frameworks^2^. To state this, let us consider a forward process *λ_τ_* (0 ≤ *τ* ≤ *t*), and a space-time trajectory *l* of a polymer chain from a specific configuration (microstate) *l*_0_ to another *l_t_* during *t*. We also consider the time reversed conjugate *l*′ of the path *l*, *i.e.*, 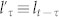 for 0 ≤ *τ* ≤ *t* under the reverse process 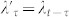. Here, we set the initial probability density for the reversed process *p*′(*x*, 0) as the final probability density for the forward process *p*(*x*, *t*) so that we have: 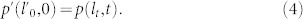
Now, the condition for microscopic reversibility^8^,^22^ reads as follows: 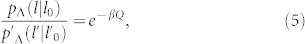
where *Q* is heat flowing into the system, *β* = 1/*k*_B_*T* (*k*_B_: the Boltzmann constant), *p*_Λ_(*l*|*l*_0_) is the conditional path probability in the sample space Λ, and 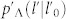 is that for the reverse process. We note that 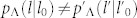 in general.

Given the above setup, we construct “local non-equilibrium thermodynamics”, where locally-defined entropy, information, and free energy are true thermodynamic potentials for each microstate, in sharp contrast with “stochastic thermodynamics”, where they are trajectory-dependent.

## Results

### Local functions as state functions of Λ*^x^*

Firstly, we prove that various local functions defined at each phase-space point *x* at time *t* are actually state functions of the newly introduced ensemble Λ*^x^*. Let us consider a local form of information theoretic Shannon entropy (so called stochastic entropy)^8^,^12^. To show that this entropy is the property of the ensemble Λ*^x^*, we define a quantity for Λ*^x^*: 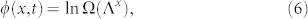
which we shall call *information*. As a reference value let *φ*_0_ be the maximally attainable value of information, *i.e.*, *φ*_0_ = ln Ω(Λ) (see Fig. 2a). Then, *σ*(*x*, *t*) ≡ *k*_B_(*φ*_0_ − *φ*(*x*, *t*)) becomes 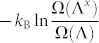. Since the argument of the logarithm is simply the ratio of the total weight of paths in Λ*^x^* to that of Λ, which becomes *p*(*x*, *t*), *σ*(*x*, *t*) is identical to the local Shannon entropy. Thus, stochastic entropy *σ*(*x*, *t*) can be viewed as a property of the ensemble Λ*^x^* as desired. Here we emphasize that the local entropy is now defined through a means of counting. We remark that *φ*(*x*, *t*) may be interpreted as the information on the occurrence of the microstate (*i.e.*, the polymer configuration), entropy *σ*(*x*, *t*) as *lost information* upon specifying a particular microstate *x*. Thus, the sum of information *φ*(*x*, *t*) and entropy *σ*(*x*, *t*) conserves *locally*. Hence, any theory for information could be interpreted using entropy and vice versa, and we will select one depending on the context. In this picture, every microstate of a system carries information, and thus we will consider information gain/consumption of each microstate but not information measurement/erasure procedures formulated in terms of mutual information^23^,^24^,^25^,^26^. Now we define a free-energy-like quantity as follows: 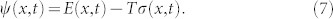
Note that *σ*(*x*, *t*) = *k*_B_(*φ*_0_ − *φ*(*x*, *t*)). Thus, local free energy *ψ*(*x*, *t*) enables us to treat the information and energy of each microstate on an equal footing since *ψ*(*x*, *t*) is nothing but the sum of energy *E*(*x*, *t*) and information *φ*(*x*, *t*) adjusted by the reference value *φ*_0_ and scaled by *k*_B_*T*.

It was shown by Hummer and Szabo^14^ that energy *E*(*x*, *t*) could be represented as weighted average of work functions over all paths in Λ*^x^*. In detail, they derived the following relation using the Feynmann-Kac formula: 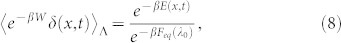
where the bracket indicates an average over all paths in Λ, *δ*(*x*, *t*) is the Dirac-delta function at a microstate *x* and time *t*, and *F_eq_*(*λ*_0_) is the equilibrium free energy of a macrostate *λ*_0_. Due to the end-point conditioning by *δ*(*x*, *t*), only the paths in Λ*^x^* among Λ contribute to the value of the left-hand side of this relation. Thus, equation (8) shows that energy *E*(*x*, *t*) is to be a property of the ensemble Λ*^x^*. Accordingly, the local free energy *ψ*(*x*, *t*) is also a property of Λ*^x^* as desired (see Supplementary Information A). We note that the average of *ψ*(*x*, *t*) over microstates is known as the *effective free energy* of a macrostate, and relations between the effective free energy and relative entropy of macrostates have been investigated in Ref. 27.

### Roles of local free energy *ψ*(*x*, *t*) within Λ*^x^*

Now, we derive fundamental relations for each non-equilibrium microstate, which share their mathematical forms with those of classical and modern theories.

Firstly, we show that the newly introduced local free energy *ψ*(*x*, *t*) is a critical quantity in determining the non-equilibrium probability of each microstate *x*. As above, we explicitly calculate the ratio of the total weight of paths in Λ*^x^* to that in Λ (see Supplementary Information B). Then, from equation (6) and equation (7), we have the following: 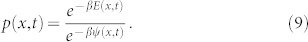
This relation shows that local free energy *ψ*(*x*, *t*) plays a role analogous to equilibrium free energy for non-equilibrium microstates. We stress that *p*(*x*, *t*) is expressed solely by (micro-)state functions (*i.e.*, true thermodynamic potentials) of the ensemble Λ*^x^*, which is independent of specific paths to realize a microstate *x*. Here it may be worth noting that the structure of our theory is not dependent on the resolution used for specifying a microstate (see Methods). In the literature, equation (9) appears implicitly either from definitions of path-dependent stochastic entropy and free energy, or mixed with work functions (path functions) if an ensemble is considered^28^,^29^. For the latter, *p*(*x*, *t*) could be written in the above simple form as a property of the ensemble Λ*^x^* only if the relation between *ψ*(*x*, *t*) and work functions were resolved as shown later in equation (13).

Secondly, we prove a *local* version of the Crooks relation. The original Crooks relation is a core equation that can generate various modern fluctuation theorems within Λ focusing on implementing a macrostate *λ_t_* from an initial ensemble^28^. The *local* Crooks relation that we prove holds while implementing a single microstate, and thus it can generate various fluctuation theorems within Λ*^x^* as will be shown below. Using equation (5) and equation (9), we have 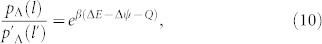
where Δ*E* = *E*(*l_t_*, *t*) − *E*(*l*_0_, 0), and Δ*ψ* = *ψ*(*l_t_*, *t*) − *ψ*(*l*_0_, 0). Applying the first law as in equation (3), we have 
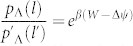
. Now we consider path probabilities within Λ*^x^* so that 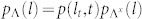 and 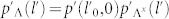. Due to equation (4), we obtain the following *local* Crooks relation: 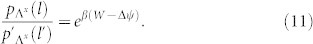
This relation implies that fluctuation theorems for (realizing) a macrostate remain the same even if we focus on a single microstate. Most importantly, the local Crooks relation provides a physical interpretation for local free energy *ψ*(*x*, *t*). To see this, we integrate equation (11) over the ensemble Λ*^x^*, giving: 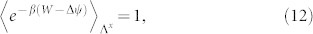
where the bracket indicates an average taken over all paths to *x* at time *t*. If we assume that *p*(*x*, 0) is the Boltzmann-Gibbs distribution (then, we have *ψ*(*x*, 0) = *F_eq_*(*λ*_0_) for all *x* (see equation (15) below)), we may rewrite equation (12) as follows: 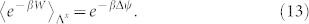
This is nothing but the local form of Jarzynski's relation, expressed for each microstate, revealing that the local free energy *ψ*(*x*, *t*) encodes (regulates) work contents for realizing a single microstate *x* from an initial ensemble. This role of *ψ*(*x*, *t*) is analogous to that of equilibrium free energy *F_eq_*(*λ_t_*), as expected. As a corollary, we have 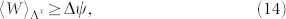
which shows that average efficiency of the conversion from work to *ψ* is less than 100%, indicating that the second law of thermodynamics holds even within the ensemble of realizations of each microstate *x*. It should be noted that local ensemble Λ*^x^* is critical to reveal this role of *ψ*(*x*, *t*): This term would otherwise be integrated out to give equilibrium free energy (see equation (15)). We remark that equation (13) is a highly desired generalization of Landauer's principle, which quantifies fluctuations in erasure processes^30^,^31^ (see Examples and also Discussion).

### Roles of local free energy *ψ*(*x*, *t*) between Λ*^x^*s

Finally, we prove two inter-microstate relations. The first relation explains how non-equilibrium work measurement could give equilibrium free energy. Since the *accessible number* of microstates, 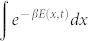, is a function of an equilibrium free energy *F_eq_*(*λ_t_*) of a state *λ_t_*, equation (9) implies the following link between the instant distribution for local free energy *ψ* and an equilibrium free energy for the corresponding macrostate: 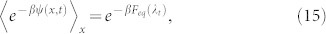
where the bracket indicates the average over all microstates (chain configurations). According to equation (13), non-equilibrium work measurement within Λ*^x^* gives non-equilibrium free energy *ψ*(*x*, *t*). Thus if the measurement is done over all paths in Λ, it corresponds to taking the average of *ψ*, which gives equilibrium free energy as expressed in equation (15). As a corollary, we have 〈*ψ*(*x*, *t*)〉*_x_* ≥ *F_eq_*(*λ_t_*). In equilibrium, *ψ*(*x*, *t*) = *F_eq_*(*λ_t_*) holds for all *x* due to the strict convexity of the exponential function^32^. This condition together with equation (9) gives the Boltzmann-Gibbs distribution, and clearly characterizes the meaning of the “*least biased*” in an equilibrium as presented by Jaynes^33^. Boltzmann assigned each microstate an equilibrium thermal quantity: The above equilibrium condition for *ψ*(*x*, *t*) shows that this is valid only in equilibrium.

Next, we demonstrate the critical role of local free energy *ψ*(*x*, *t*) in non-equilibrium relaxation. We consider a Brownian system described by the following Langevin equation, which is a minimal prototype that contains the stochasticity: 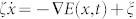, where *ζ* is the friction constant and *ξ* is the fluctuating force that satisfies the fluctuation-dissipation theorem, 〈*ξ*(*t*)*ξ*(*t*′)〉 = 2*k*_B_*Tζδ*(*t* − *t*′). Then, the probability density *p*(*x*, *t*) of this system obeys the Fokker-Planck equation as 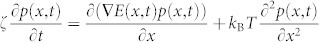. From equation (9), the time evolution of local information *φ*(*x*, *t*) is described by 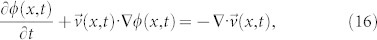
where 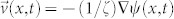 (see Supplementary Information C). We note that this flow driven by local free energy gradient should be interpreted as information flow (not entropy flow) (see Fig. 2b). When information flows from *x* to *x*′ with a loss, we may say that entropy flows with an increase to the opposite direction, *x*′ to *x*, since the sum of them is conserved locally. Thus, equation (16) tells us that for regions of high work content, *ψ* spontaneously decreases through the exchange of local information and entropy until all available sources of energy are consumed so that driving force *ψ*(*x*, *t*) becomes constant for all *x*, *i.e.*, *E*(*x*, *t*) = *Tσ*(*x*, *t*) for all *x* (up to an additive constant). In a non-equilibrium steady state, the relation 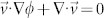 should be satisfied. Here we emphasize that the above equation is a closed form partial differential equation, which is for the information content between ensembles Λ*^x^*, *i.e.*, the quantity independent of individual paths, or (*micro*-)*state functions*. This leads to essential differences from apparently similar dynamical equations, *e.g.*, the ordinary differential evolution equation for *macroscopic* Shannon-entropy^34^ and the ordinary differential equation for entropy production *along stochastic paths*^12^.

### Examples

We present two examples of non-equilibrium systems where the concept of local free energy allows elegant representation of all relevant phenomena.

In the first example, we will see that local free energy *ψ*(*x*, *t*) provides a new insight for understanding the cyclic behaviour of the famous Szilard engine^35^. In step (1), a single particle in a box of volume *V* in contact with a heat bath of temperature *T* is in equilibrium. For analysis, we will split the phase-space of the particle into two: the left-half of the box and the right-half denoted as *L* and *R*, respectively, and use this coarse-grained phase-space (see Methods). Let *β* = 1 for a while for simplicity. Then, the free energy of the system is *F_eq_* = − ln *Z*, where 
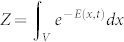
. In step (2), a partition is inserted in the middle of the box. Before measurement, the local free energy of the left and the right would be the same, *ψ*(*L*) = *ψ*(*R*) = − ln *Z*_1_ − ln 2, where 
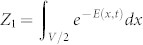
 (see equation (21)). Note that the local free energy is not different from the initial equilibrium free energy at this point, *i.e.*, *ψ*(*L*) = *ψ*(*R*) = *F_eq_*. In step (3), we measure where the particle is, and assume that it was left without loss of generality. Then, we would have *ψ*(*L*) = − ln *Z*_1_. Since *ψ* is a true thermodynamic potential, independent of paths realizing a state *L* as shown in equation (13), we can deduce that work should be consumed upon measurement such that 〈*W*〉*_L_* ≥ Δ*ψ* = ln 2 on average (equation (14)). In step (4), we link a weight in accordance with the observation in step (3) and convert heat into work by the amount of ln 2. Thus, it does not violate the second law of thermodynamics.

Here we note that a similar argument also applies to Landauer's erasure process^36^. In detail, the first and the final stage of the erasure process are identical to step (2) and step (3) of the above example of the Szilard engine, respectively. In this case, however, step (3) is achieved not by measurement but by perfectly localizing the particle to the left irrespective of its initial position (e.g., by extracting the partition separating the two halves of the box and subsequently using a piston to shift the particle to the left side of the box). In any case, consumed work is characterized by the state function *ψ*(*L*), independently of paths to realize the state. We remark that fluctuations of work (not just average work) are exactly regulated by Δ*ψ*, as expressed in equation (13).

**Figure 3:**
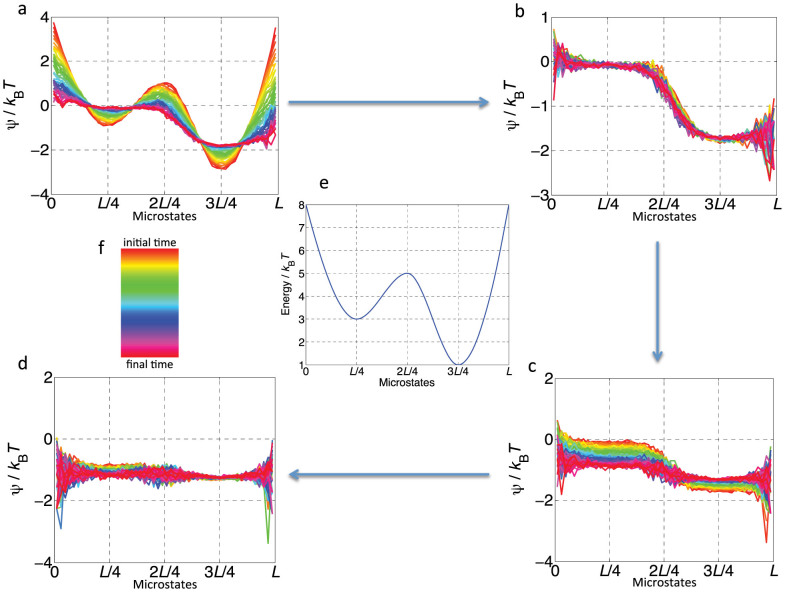
The mechanism of equilibration. Here, we describe a stochastic system in terms of local information and local free energy extracted from observations of stochastic trajectories, and show the exact mechanism of equilibration. A stochastic evolution in silico of a Brownian particle in a biased potential (e) is carried out 20,000 times with uniform initial distribution, and the time series of local free energy *ψ*(*x*, *t_i_*) profiles are shown. The colour code is shown in (f). The time-step taken is 0.01 in a dimensionless unit. (a–d) shows the profiles of free energy *ψ*(*x*, *t_i_*) from *t*_0_ to *t*_50_, from *t*_51_ to *t*_100_, from *t*_101_ to *t*_2000_, and from *t*_2001_ to *t*_4000_, respectively. (a), Due to the energy barrier in the transition state information flows into local minimum regions. As it progresses, the barrier is lowered. (b), A local equilibrium is established where the probability of microstates is proportional to the Boltzmann factor in local regions. At this stage, the bump has disappeared. (c), A global equilibration proceeds slowly compared to the first local-equilibration process. The work potential *ψ*(*x*, *t*) drives information to the right region. (d), A global equilibrium is established. Details of the simulation and results with different initial conditions are reported in Supplementary Information D.

In the second example, we illustrate how our theory converts raw data collected from fluctuating degrees of freedom into a useful set of state functions as well as the important role of local free energy *ψ*(*x*, *t*) in non-equilibrium relaxation, both by means of Brownian dynamics simulations (see Supplementary Information D). We consider a Brownian particle under a tilted bi-stable potential (Fig. 3e), which has a uniform distribution at time *t* = 0. We repeated the in-silico experiment, counted the number of particles for each partitioned bin at each time, and calculated local information and free energy. Recall that information is the accessible number of paths to a point *x*. Due to the imposed initial condition of constant local information, the local free energy *ψ* initially has the same shape as the energy *E*(*x*, *t*) over all microstates (Fig. 3a). We observe that it is *ψ* that drives the information flow to establish local equilibrium as an intermediate step (Fig. 3b) until global equilibrium is reached (Fig. 3d). This is an illustration of an exact mechanism for non-equilibrium relaxation (as expressed by equation (16)), which, to our knowledge, has not yet been achieved (see the legends of Fig. 3 and Supplementary Information D).

## Discussion

We have identified a new ensemble for each microstate, and revealed the important roles played by local free energy *ψ*(*x*, *t*) of the new ensemble. Most importantly, *ψ*(*x*, *t*) turns out to be a key quantity for generalizations of the Boltzmann-Gibbs distribution and Landauer's principle to include arbitrary fluctuations, and controls non-equilibrium relaxation. In the literature, related equations to this manuscript appear implicitly in various forms without awareness of the role of the local free energy *ψ*(*x*, *t*). For example, the most representative form for the generalized Boltzmann-Gibbs distribution appears in the literature^28^,^29^ as follows: 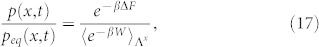
where *p_eq_*(*x*, *t*) is the Boltzmann-Gibbs distribution, Δ*F* = *F*(*λ_t_*) − *F*(*λ*_0_) that is the difference between equilibrium free energy. This relation is a combined form of equations (9), (13), and a corollary of (15). In Ref. 37, equation (9) arises simply from the definitions of *path-dependent* entropy and free energy without a significant relationship between *ψ*(*x*, *t*) and work as given in equation (13). In Ref. 31, fluctuations in Landauer's principle have been investigated using equation (17) to find Landauer's bound without awareness of the role, *i.e.*, the exact quantification of the fluctuation, played by *ψ*(*x*, *t*). Very recently, symmetry-breaking energetics (*e.g.*, for a Brownian particle that is experiencing a switching from a single-well to a double-well potential and is then confined in one well resulting in loss of ergodicity) has been investigated in Ref. 38, and it has been shown that 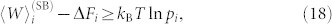
where 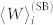 is the average work required for symmetry-breaking, Δ*F_i_* = *F_i_* − *F_eq_*(*λ*_0_), where *F_i_* satisfies 
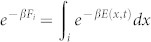
 and is called conformational free energy of the trapped region *i* of the phase-space, and *p_i_* is the probability of trapping in the region *i*. If we rewrite equation (18) using the coarse-grained phase-space notation (see Methods) and equation (22), we have 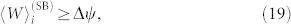
where Δ*ψ* = *ψ*(*i*, *t*) − *F_eq_*(*λ*_0_). This relation is identical to equation (14), a corollary of our main theorem, equation (13), indicating that local free energy *ψ* regulates fluctuations in symmetry-breaking energetics. Overall, these examples indicate the universal importance of local free energy *ψ*(*x*, *t*) in the thermodynamic expressions.

The second issue we discuss concerns structural similarity between an equilibrium macrostate and a non-equilibrium microstate equipped with the new ensemble Λ*^x^*. We showed that the Boltzmann-Gibbs distribution and Jarzynski's relation, which hold for (implementing) a macrostate, can be expressed in a similar manner while implementing a non-equilibrium microstate. Moreover, local functions are well-defined state functions of the new ensemble Λ*^x^*, *i.e.*, true thermodynamic potentials that are independent of paths of realizing a (micro-)state, playing similar roles to equilibrium macrostate functions like free energy. If we pursue this direction further, we may borrow various notions from equilibrium thermodynamics. For example, we may define non-equilibrium temperature locally as *dE*(*x*, *t*)/*dσ*(*x*, *t*). In principle, this approach enables us to transfer all the techniques of equilibrium thermodynamics to analyse non-equilibrium local objects. We believe that there is significant value in investigating the usefulness of such an approach in non-equilibrium systems.

## Methods

### Practical definition of a microstate

Here we briefly mention a subtlety associated with the definition of a microstate by using a polymer configuration as its example. When we try to specify a configuration of a polymer chain at time *t*, there is always a problem of the space-time resolution that is used to distinguish different microstates. This is particularly the case for experiments and simulations. This resolution problem can be resolved by a coarse-grained partition of the phase space. Let 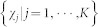 be a partition of the phase space with *K* non-overlapping subsets *χ_j_*. We may regard *χ_j_* as a coarse-grained microstate. The information of *χ_j_* is defined as follows: 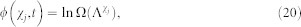
where 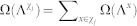. Then, we apply the previous argument and obtain the non-equilibrium probability of *χ_j_*, 
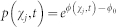
. In this case, we shall define the free energy of *χ_j_* as follows: 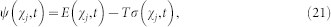
where *σ*(*χ_j_*, *t*) = *k*_B_(*φ*_0_ − *φ*(*χ_j_*, *t*)), identical to stochastic entropy, and *E*(*χ_j_*, *t*) is defined such that 
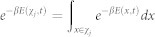
. Then, we have 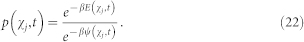
We note two things here: Firstly, the structure of our theory is invariant under the transformation of a microstate *x* to a coarse-grained one *χ_j_*. Secondly, when we do not divide fluctuating degrees of freedom, our theory reduces to global theorems in a natural way. For example, let a single coarse-grained microstate *χ* be the set of all fluctuating degrees of freedom. Then *ψ*(*χ*, *t*) becomes *F_eq_*(*λ_t_*) from equation (21). We see immediately that equations (11)–(14) in the main text reduce to the corresponding known global relations, and equations (9) and (15) reduce to trivial identities. Similarly, it may be instructive to apply the same framework to an equilibrium situation, where a system is in a stationary state with thermal fluctuations. Even in an equilibrium situation, we can think of an initial state and a state at time *t*. Thus we can still define *φ*(*x*, *t*) and *ψ*(*x*, *t*). Our intuition immediately tells us that *σ*(*x*, *t*) and *ψ*(*x*, *t*) should both be independent of *x* and *t*, suggesting that their averages are standard entropy and free energy, respectively.

### Experimental measurement

As an example, we consider a polymer chain (in a heat bath of temperature *T*) whose one end is fixed and the other end is under an external control *λ_τ_* that varies over time 0 ≤ *τ* ≤ *t* in a well-defined manner, as shown in the right panel of Fig. 1. We prepare the system initially to be sampled according to a probability density *p*(*x*, 0). Let us assume that *p*(*x*, 0) is the Boltzmann-Gibbs distribution for simplicity. We carry out the experiment by controlling *λ_τ_* during 0 ≤ *τ* ≤ *t*, and measure work *W* done on the system. If we have additional information on the final microstate, our theory converts the fluctuating details into *φ* and *ψ* (a microstate can be a coarse-grained one). We repeat the process *N* times, a large number. Let *N_x_* be the number of the experiments whose final microstate is *x*. Here we put a number to path *i* reaching microstate *x* from *i* = 1 to *N_x_* and express the work done in path *i* as *W_i_*. Then, we obtain the energy of *x* using the following equality for Λ*^x^*: 
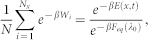
as proven in Ref. 14. Here *F_eq_*(*λ*_0_) is the equilibrium free energy of the initial *macrostate*. It is important to note that the normalization factor is *N*. If the summation is taken over all experiments, it reduces to the Jarzynski relation. We also proved that 
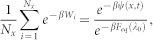
giving *ψ*(*x*, *t*). It is important that the normalization factor here is *N_x_*. These two equalities are linked by the the relation between *ψ*(*x*, *t*) and *F_eq_*(*λ_t_*) in equation (15).

## Author Contributions

L.J and H.T. conceived the work, developed the theory, and wrote the paper.

## Supplementary Material

Supplementary Information Supplementary Information
