# Supplementary material for: Local non-equilibrium thermodynamics
Source: Sci Rep. 2015 Jan 16;5:7832. doi: 10.1038/srep07832 (PMC4296294; doi:10.1038/srep07832)
Supplement: Supplementary Information [file srep07832-s1.pdf]

Supplementary Information for
"Local non-equilibrium thermodynamics"

Lee Jinwoo[1] and Hajime Tanaka[2]

[1] *Department of Mathematics, Kwangwoon University, 20 Kwangwoon-ro, Nowon-gu, Seoul 139-701, Korea*

[2] *Institute of Industrial Science, University of Tokyo, 4-6-1 Komaba, Meguro-ku, Tokyo 153-8505, Japan*

A. Illustration of local ψ.

Here, we consider a Brownian particle in a box in contact with a heat bath of temperature T and block it incompletely as shown in Fig. S1. Let Λ^A be all the paths from an initial ensemble to A at time t. We define *the accessible number* (or the total weight) $\Omega(\Lambda^A)$ of paths in Λ^A by $\Omega(\Lambda^A) = \sum_{l\in\Lambda^A} g(l)$, where $g(l)$ is a weight of a path l such that $g(l)$ is proportional to the probability of path l (see the main text, or Section B below). The information content $\phi(A,t)$ is defined by $\phi(A,t) = \ln\Omega(A,t)$. As a reference value ϕ_0 is defined by the logarithm of the total weight of all paths, *i.e.*, $\phi_0 = \ln\Omega(\Lambda)$, where Λ is the set of all paths. Now, the presence of the partial wall increases the accessible number (or the total weight) $\Omega(\Lambda^A)$ of paths to A, so does information content $\phi(A,t)$, and thus the local free energy $\psi(A,t)$. Note that the local free energy is defined by $\psi(A,t) = E(A,t) - T\sigma(A,t)$, where $E(A,t)$ is the energy of the microstate A at time t and $\sigma(A,t) = k_\mathrm{B}(\phi_0 - \phi(A,t))$. Similarly, $\psi(B,t)$ would be decreased. As we will see from the main text, ψ is the work content. Thus, we could extract work from the increase of $\psi(A,t)$ by linking a weight to the partition during non-equilibrium evolution of the particle. We also note that the thermodynamical asymmetry between A and B is due to the initial condition, indicating the importance of initial conditions in non-equilibrium [1].

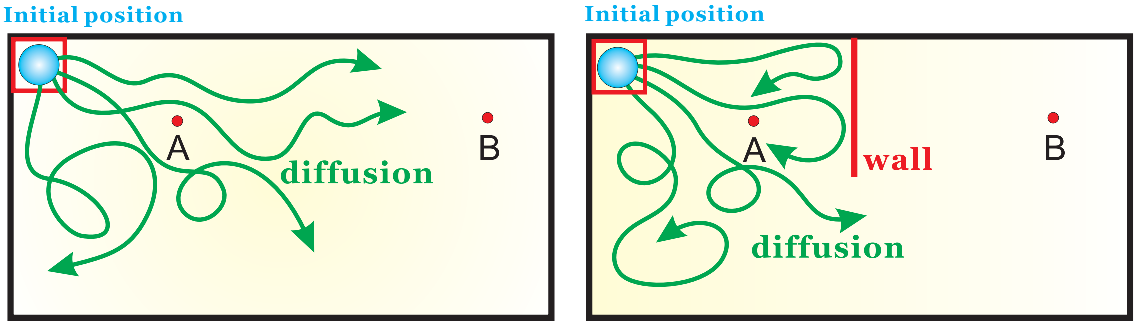

Fig. S1. **An incompletely-blocked particle.** A colloidal particle is released at $t = -\epsilon$ for a small $\epsilon > 0$ from the upper-left corner so that it has non-vanishing probability at $t = 0$, and moves stochastically. We repeat the experiment, and consider to block the particle by a partition incompletely as shown in the right panel. During non-equilibrium evolution, the presence of the wall increases the information content of the ensemble Λ^A of A resulting in an increase of the local free energy $\psi(A, t)$ for some t. Accordingly, $\psi(B, t)$ would be decreased.

B. Derivation of Eq. (9).

Let Λ be the set of all possible space-time trajectories of an experiment. When we count *the accessible number* of paths, we assign each path a weight of the form $g(l) \equiv e^{-s(l)}$, where $s(l)$ is such that the probability of path l is represented as $p(l) \propto e^{-s(l)}$, so that less probable paths are to be less counted. Then, a probability of path l in a sample space X would be $p(l) = g(l)/\sum_{l\in X} g(l)$. It would be convenient to deal with a trajectory in a discrete approximation as a set of states x_i in consecutive times t_i for $i = 0, \cdots, n$. Let us denote the set of trajectories that pass x_k at time t_k as $\Lambda(x_k, t_k)$ for some integer k (see Fig. S2). We will calculate the ratio of the number of paths in $\Lambda(x_k, t_k)$ to the number of all possible trajectories in Λ. Since the probability of path l is $p(l) = g(l)/\sum_{l\in\Lambda} g(l)$, the ratio becomes $\sum_{l\in\Lambda(x_k,t_k)} p(l)$. Then, the ratio would be approximated as $\sum_{x_0} \cdots \sum'_{x_k} \cdots \sum_{x_n} p(x_0, \cdots, x_k, \cdots, x_n)$, where each sum is taken over the phase-space points at time t_i, and $\sum'_{x_k}$ means that we omit the sum over the phase-space points at time t_k. It is important to omit that sum at time t_k, otherwise the result would be 1. By the law of total probability the sum reduces to $p(x_k, t_k)$.

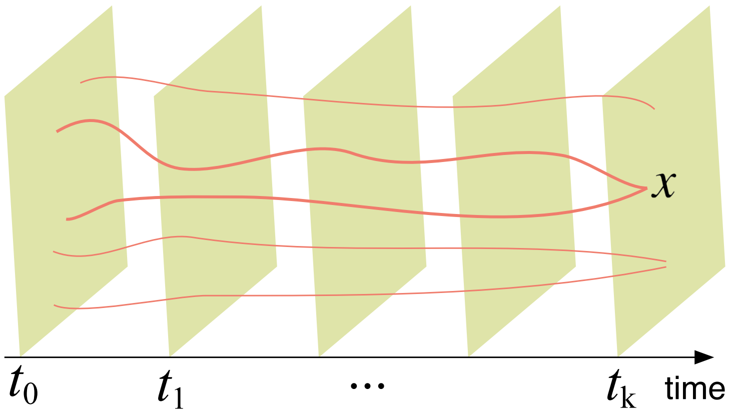

Fig. S2. **A microstate as an ensemble of trajectories to the phase-space point.** Each space in green represents the phase space of a system (excluding momentum variables for simplicity) at time t_i. Each trajectory shown schematically represents a stochastic evolution of the phase-space points. Among all possible trajectories, we focus on those paths (thick lines) that reach a specific point x in the phase space at time t_k.

C. Derivation of dynamic rules.

We consider the Langevin equation, which is a minimal prototype that contains an essence of stochasticity: $\zeta \dot{x} = -\nabla E(x,t) + \xi$, where $E(x,t)$ is energy of a microstate x at time t, ζ is the friction constant and ξ is the fluctuating force that satisfies the fluctuation-dissipation theorem, $\langle \xi(t)\xi(t')\rangle = 2k_\mathrm{B}T\zeta\delta(t-t')$. The probability density $p(x,t)$ of a system under the overdamped Langevin equation obeys the Fokker-Planck equation as

$$\zeta \frac{\partial p(x,t)}{\partial t} = \frac{\partial \left(\nabla E(x,t) p(x,t)\right)}{\partial x} + k_\mathrm{B}T \frac{\partial^2 p(x,t)}{\partial x^2}.$$

From $p(x,t) = e^{\phi(x,t)-\phi_0}$ (see the main text or Sections A and B above), we have $\frac{\partial p}{\partial x} = p\frac{\partial \phi}{\partial x}$ and $\frac{\partial^2 p}{\partial x^2} = p\left(\frac{\partial \phi}{\partial x}\right)^2 + p\frac{\partial^2 \phi}{\partial x^2}$. Substituting these relations into the Fokker-Planck equation, and dividing by $p(x,t)$, we obtain the following equation:

$$\zeta \frac{\partial \phi(x,t)}{\partial t} = \frac{\partial E(x,t)}{\partial x}\frac{\partial \phi(x,t)}{\partial x} + k_\mathrm{B}T\left(\frac{\partial \phi(x,t)}{\partial x}\right)^2 + \frac{\partial^2 E(x,t)}{\partial x^2} + k_\mathrm{B}T\frac{\partial^2 \phi(x,t)}{\partial x^2}.$$

Here the first two terms in the right-hand side may be written as $\frac{\partial}{\partial x}\left(E(x,t) + k_\mathrm{B}T\phi(x,t)\right)\frac{\partial \phi(x,t)}{\partial x}$, and the second two terms as $\frac{\partial^2}{\partial x^2}\left(E(x,t) + k_\mathrm{B}T\phi(x,t)\right)$. Noting that the sum in the paren-

thesis is just $\psi(x,t)$ (see the main text or Section A above), we have

$$\frac{\partial \phi(x,t)}{\partial t} - \frac{1}{\zeta}\nabla\psi(x,t)\cdot\nabla\phi(x,t) = \frac{1}{\zeta}\nabla^2\psi(x,t),$$

where ∇^2 denotes the Laplacian. This is a non-linear convection-diffusion equation with a source term. It determines the dynamics of ϕ and ψ completely, given initial and boundary conditions, where the energetic cost of the information flow is mediated by heat with less than 100% efficiency resulting in a net loss of information, andthus a net increase of entropy.

D. Details of in-silico experiments.

We carried out in-silico experiments of the Brownian movement of a particle. We numerically solve the following overdamped Langevin equation:

$$\zeta\dot{x} = -\nabla E(x,t) + \xi,$$

where $E(x,t)$ is energy of a microstate x, and thermal fluctuation ξ satisfies $\langle\xi(t)\xi(t')\rangle = 2k_{\mathrm{B}}T\zeta\delta(t-t')$. Here we set $\zeta = 1$ for simplicity. Then, after discretization, we have

$$x(t_{i+1}) = x(t_i) - \nabla E(x(t_i), t_i)\epsilon + \sqrt{2k_{\mathrm{B}}T\epsilon}\,r(t_i),$$

where $\epsilon = t_{i+1} - t_i$ and $r(t_i)$ is a random number drawn from the standard normal distribution. In our simulations, we set the mobility to unity, and $k_{\mathrm{B}}T$ to 2 by rescaling. We constrained the particle by setting the reflecting boundaries at $x = 0$ and $x = L$ ($L = 10$). We used $\epsilon = 0.01$. The domain is partitioned into 50 bins, and we counted the number of particles for each bin at each time to obtain the graphs of information and free energy.

Figure S3 shows the profiles of information and free energy when the initial condition is set to $p(x,0) = \delta(x - L/4)$. In this case, there is no barrier in $\psi(x,t)$ although there is an energy barrier. Thus, the local equilibrium is established quickly in the initial stage. Then the free energy ψ drives information ϕ to the right region until reaching the global equilibrium. During this second stage, the flow continues without breaking the established local equilibrium. Note that the profile of information evolves from the hat shape to the shape that exactly compensates the energy profile up to an additive constant.

Now we put initially the particle at the location of the global minimum of energy, *i.e.* $p(x,0) = \delta(x - 3L/4)$, and Fig. S4 shows the profiles of information and free energy over microstates. There is no barrier in the free energy profile ψ. First, the local equilibrium is established quickly, and the flow of information ϕ continues towards the left region although

the speed is much slower than the local equilibration process. Second, the flow of information ϕ continues until the global equilibrium is established without breaking the local equilibrium. In this case again, the profile of information at the equilibrium compensates exactly the energy profile up to an additive constant.

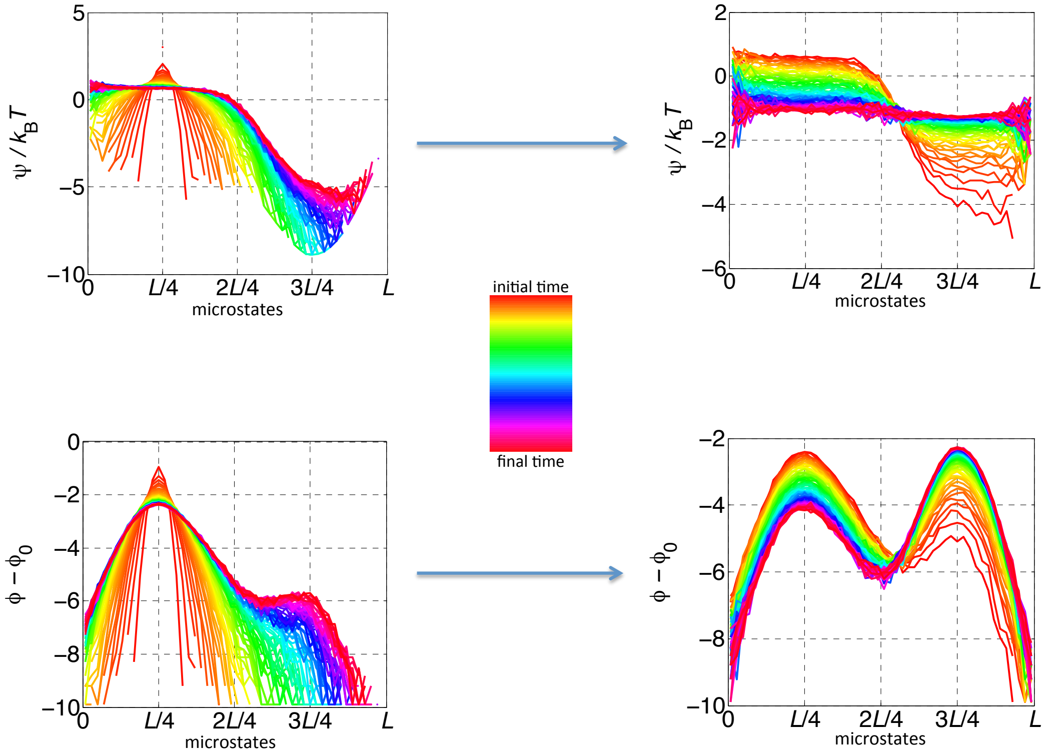

Fig. S3. **The mechanism of equilibration: case I.** Here we put the particle only at $x = L/4$ during the repetition of the simulation so that $p(x,0) = \delta(x - L/4)$. The other conditions are the same as the case in the main text. The time series of free energy over microstates is shown in the upper panel, and those of information in the lower panel. The profiles are from t_0 to t_{100} for the left figures, and t_{101} to t_{4000} for the right figures. Due to the initial condition, there is no free energy barrier in this case. The process towards local equilibrium is very quick. The global equilibrium proceeds without breaking the established local equilibrium.

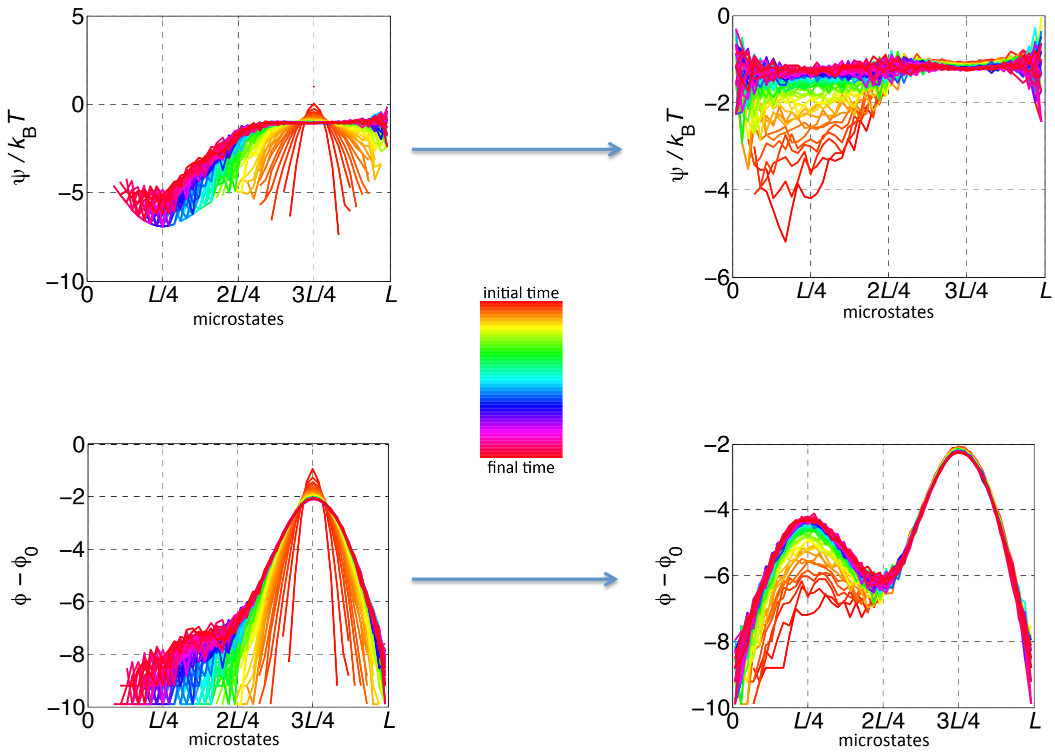

Fig. S4. **The mechanism of equilibration: case II.** Here we put the particle only at $x = 3L/4$ during the repetition of the simulation so that $p(x, 0) = \delta(x - 3L/4)$. The other conditions are the same as the case in the main text. The time series of free energy over microstates is shown in the upper panel, and those of information in the lower panel. The profiles are from t_0 to t_{100} for the left figures, and t_{101} to t_{4000} for the right figures. Due to the initial condition, there is no free energy barrier in this case. The process towards local equilibrium is very quick. The global equilibrium proceeds without breaking the established local equilibrium.

[1] Jarzynski, C. Equalities and inequalities: Irreversibility and the second law of thermodynamics at the nanoscale. *Annu. Rev. Codens. Matter Phys.* **2**, 329–51 (2011).
